# Efficacy of liquid biopsy for disease monitoring and early prediction of tumor progression in EGFR mutation-positive non-small cell lung cancer

**DOI:** 10.1371/journal.pone.0267362

**Published:** 2022-04-28

**Authors:** Hsiang-Ling Ho, Yuqiu Jiang, Chi-Lu Chiang, Sylwia Karwowska, Ranga Yerram, Keerti Sharma, Sidney Scudder, Chao-Hua Chiu, Chun-Ming Tsai, John F. Palma, Abha Sharma, Teh-Ying Chou

**Affiliations:** 1 Department of Pathology and Laboratory Medicine, Taipei Veterans General Hospital, Taipei, Taiwan; 2 Clinical Development and Medical Affairs, Roche Diagnostic Solutions, Pleasanton, California, United States of America; 3 Division of Thoracic Oncology, Department of Chest Medicine, Taipei Veterans General Hospital, Taipei, Taiwan; College of Medicine, National Cheng Kung University, Tainan Taiwan, TAIWAN

## Abstract

15–40% of non-small cell lung cancer (NSCLC) patients harbor epidermal growth factor receptor (EGFR)-sensitizing mutations. Tyrosine kinase inhibitors (TKIs) provide significant clinical benefit in this population, yet all patients will ultimately progress. Liquid biopsy can reliably identify somatic tumor-associated EGFR mutations in plasma. This study aimed to assess the feasibility and value of the quantitative assessment of EGFR driver mutations in plasma in EGFR-mutated NSCLC patients treated with EGFR-TKIs as a tool to evaluate therapeutic response to TKIs and monitor for disease progression. The study included 136 patients with tissue biopsy-confirmed EGFR-sensitizing, mutation-positive lung adenocarcinoma with plasma collected prior to TKI treatment and at least two post-initiation TKI treatment/follow-up blood samples. Plasma samples were tested with the cobas^®^ EGFR Mutation Test v2 (cobas EGFR Test), and semi-quantitative index (SQI) values for each identified mutation were reported by the assay software. The most common baseline EGFR mutations detected in tissue were L858R (53.7%) and exon 19 deletion (39.7%). Plasma cell-free DNA analysis detected EGFR mutations in 74% of the baseline samples. Objective response rate by RECIST 1.1 was achieved in 72% of patients, while 93% had a molecular response (defined as disappearance of the EGFR mutation from plasma). 83% of patients had molecular progression (MP; 1.5X SQI increase or new T790M mutation), and 82% who had a clinical response had clinical progression. On average, MP occurred 42 days prior to clinical progression. Patients who progressed while on first-line TKI showed MP of the original EGFR-sensitizing mutations prior to the emergence of a T790M mutation, which was detected in 27% of the EGFR plasma-positive patients. Longitudinal monitoring of EGFR mutational load in plasma is feasible and can predict both response and clinical progression in EGFR-mutated NSCLC patients treated with EGFR-TKIs, as well as detect treatment-emergent EGFR mutations.

## Introduction

Activating mutations within the tyrosine kinase domain of the epidermal growth factor receptor (EGFR) are present in non-squamous non-small cell lung cancer (NSCLC), ranging from 10%–15% in Caucasian to 40%–50% in Asian populations [1–5]. The most common alterations include a series of exon 19 deletions (Ex19Dels) and the exon 21 L858R point mutation. The presence of these mutations results in constitutive activation of EGFR, driving the oncogenic process, and is associated with sensitivity to EGFR tyrosine kinase inhibitors (TKIs). EGFR TKI therapy leads to significantly prolonged progression-free survival (PFS) and overall survival compared with platinum-containing doublet chemotherapy [6]. Although patients achieve great benefit from anti-EGFR therapies, all patients eventually develop resistance to the treatment with progression of their disease.

Studies have demonstrated that mutational profiles change during the course of treatment [7–9], which would be challenging to assess through repeat tissue analysis alone. As tissue biopsies represent only a small portion of one tumor deposit, it would be difficult to investigate spatial and temporal tumor mutational heterogeneity [10–12]. Liquid biopsy is a non-invasive way to identify somatic mutations from circulating tumor (ctDNA) for molecular profiling [13]. Theoretically, it offers a real-time assessment of total-body molecular tumor genotype assuming all tumor deposits shed DNA into the bloodstream. Ease of sampling also provides the opportunity for frequent mutational evaluation and monitoring of mutational load over time [14]. The cobas^®^ EGFR Mutation Test v2 (cobas EGFR Test) in plasma samples can assess EGFR mutational load at diagnosis and be used to follow patients with EGFR-sensitizing mutations to evaluate longitudinal changes in EGFR mutation status during TKI treatment [8]. It also can detect T790M, the predominant resistance mutation to first- and second-generation TKIs, which occurs in approximately 50% of patients who progress on EGFR TKI therapy. This test also offers a unique tool called the semi-quantitative index (SQI), which is a relative measure of the amount of mutant cell-free DNA (cfDNA) in a sample for a given patient that can be used to monitor/track/measure the evolution of EGFR mutations over time [15].

In this study, we demonstrate that monitoring EGFR mutations during TKI treatment could indicate response to therapy and predict subsequent progression of disease. We also examine increasing levels of the patient’s EGFR-sensitizing mutations and/or the appearance of a new T790M mutation as an indication of resistance to therapy.

## Materials and methods

### Patients

**Fig 1:**
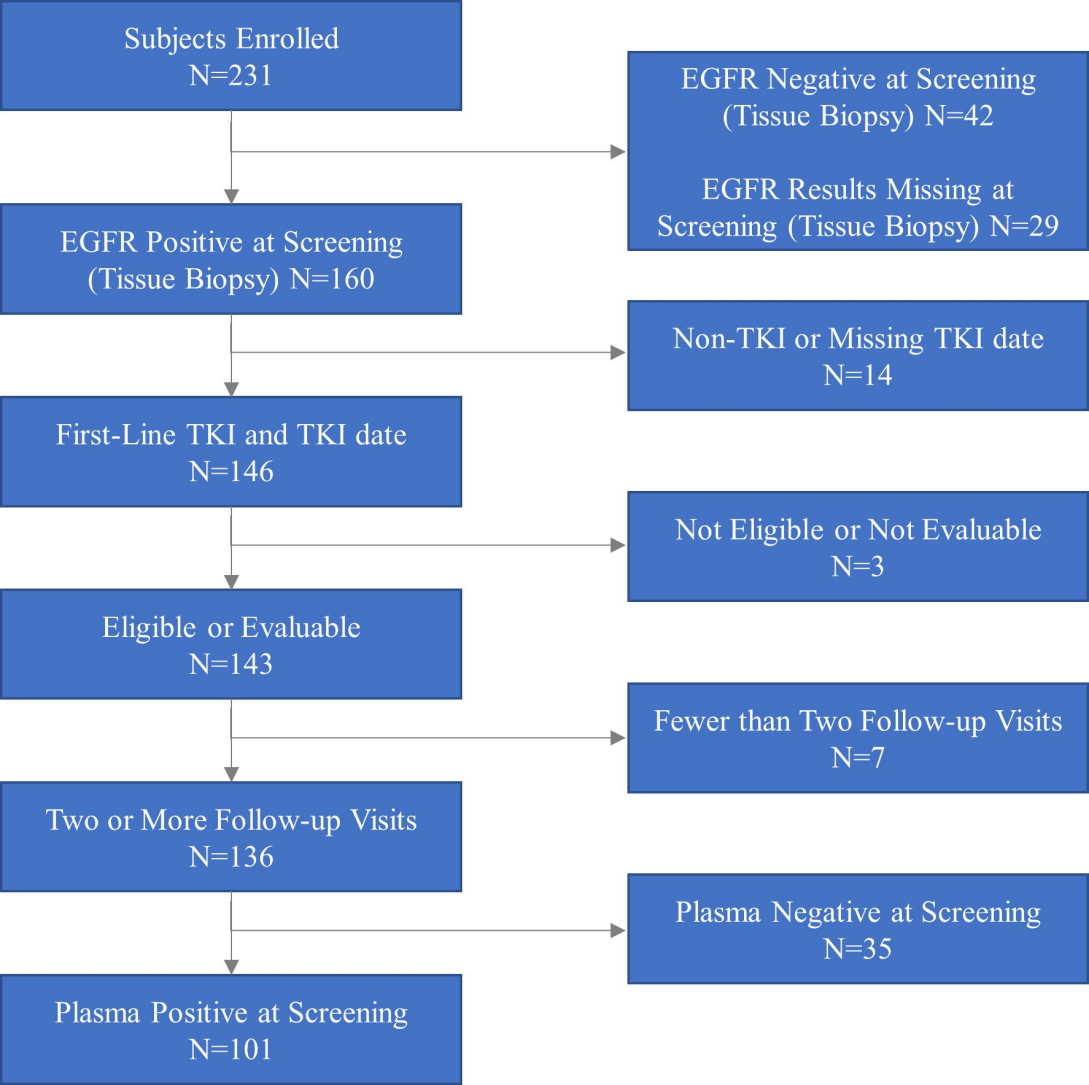
Patient flow diagram. “Non TKI” means that patients were not treated with anti-EGFR TKIs due to clinical reasons. “Missing TKI date”–we excluded patients with the drug administration date missing or not available in the database. “First line TKI and TKI date” means EGFR TKI as first-line treatment was given and date of first-line TKI was available. “Eligible or evaluable”–one patient withdrew consent during the study.

There were 231 NSCLC patients from a single clinical site in Taiwan prospectively screened for this study. This analysis was focused on those patients who were EGFR mutation tissue positive by the cobas EGFR Test, were treated with first-line anti-EGFR TKIs, and had at least two follow-up visits, as shown in the flow chart (Fig 1). At the time of this analysis, the average follow-up time was 279 days. Written informed consent was obtained from all patients participating in the study. Approval from institutional Ethics Committees was obtained for all patients. The study was approved by the Institutional Review Board, Taipei Veterans General Hospital (TPEVGH) (IRB number: 2015-03-006A) and was conducted in accordance with the Declaration of Helsinki. Additional information regarding the ethical, cultural, and scientific considerations specific to inclusivity in global research is included in the S1 Checklist.

### Tumor tissue and blood sample collection and DNA extraction

Tumor tissue samples collected at the time of diagnosis of advanced/metastatic disease, by resection or biopsy, were required before the start of first-line TKI. Blood samples were collected at baseline before the start of TKI therapy and subsequently at the scheduled 6-week intervals through disease progression. At each collection, blood samples were collected in BD Vacutainer K2EDTA tubes (BD Biosciences, San Jose, CA, USA) and subjected locally to plasma separation within 30 minutes. After centrifugation at 1000 rpm for 15 minutes, the plasma was aliquoted and stored at −80°C until use. DNA was extracted from tumor tissue and plasma using either the cobas DNA or cfDNA Sample Preparation Kits (Roche Molecular Systems, Pleasanton, CA, USA), according to the manufacturer’s instructions.

### Assessment of EGFR mutations from tissue samples and plasma DNA

EGFR mutation analysis in tissue and plasma samples used the cobas EGFR Test, an allele-specific polymerase chain reaction (PCR) assay, designed to detect 42 EGFR mutations: G719A/C/S in exon 18 (G719X); 29 deletions in exon 19; S768I, T790M, five insertions in exon 20; and L858R and L861Q in exon 21. Analysis was confirmed by negative and positive controls contained in the kit. The PCR reactions were performed on the cobas z 480 analyzer with EGFR Analysis Package Software (Roche Molecular Systems, Pleasanton, CA, USA). The cobas EGFR Test includes a SQI* which reflects the mutational load in plasma for a given variant and allows for following the mutational load longitudinally in an individual patient (SQI values cannot be compared between patients). The SQI was derived from a dilution series containing known copy numbers of mutated EGFR and a fixed amount of wild-type EGFR, with the wild-type DNA serving as an internal control during real-time PCR. The SQI* is reported as an automated result from the cobas z 480 software when an EGFR mutation is detected in ctDNA.

*not available in the USA.

### Clinical and molecular response to TKI and statistical analysis

Computed tomography (CT) of the chest was performed to monitor treatment response and progression every 2–3 months per standard clinical practice. The determination of complete response, partial response, stable disease, and progressive disease was based on RECIST 1.1 criteria. Molecular response was defined as no detected EGFR mutations in plasma samples by the cobas EGFR Test in those patients in which an EGFR mutation was detected in their baseline plasma sample. Cumulative incidence method was used to estimate the time to response for both clinical and molecular response.

Molecular progression was defined as a 1.5x numerical increase in SQI values or detection of T790M by the cobas EGFR Test. Time to clinical progression and molecular progression was examined using the Kaplan-Meier method, and an unadjusted Cox proportional hazards model was used to assess the association of each category.

## Results

Two hundred and thirty-one NSCLC patients were screened and tested for EGFR mutations in tissue using the cobas EGFR Test. As indicated in Fig 1, 160 patients were tumor tissue positive for EGFR mutations. Excluding patients who were missing treatment dates, were not eligible or not evaluable for the study, or had fewer than two follow-ups, 136 patients were available for analysis. Of these 136 patients, 101 patients were EGFR mutation positive in plasma at baseline (74%). The most common EGFR mutations detected in the tissue were L858R (53.7%) and Ex19Del (39.7%) (Table 1). One patient had both Ex19Del and T790M mutations. The prevalence of L858R and Ex19Del in plasma samples was 49.5% and 44.5%, respectively. Afatinib was the most common EGFR TKI used for first-line treatment in more than 55.88% of the study population followed by erlotinib and gefitinib. There were significant differences between plasma EGFR mutation positive and negative patients in whom EGFR TKI therapy was used as first-line treatment (Table 1).

**Table 1:** Patient demographics and baseline clinical characteristics by EGFR mutation status.

Characteristics		Overall subjects (n = 136)	Plasma-positive subjects at screening (n = 101)	Plasma-negative subjects at screening (n = 35)	P-value
**Sex**	Female	76 (55.88%)	54 (53.47%)	22 (62.86%)	0.3349
	Male	60 (44.12%)	47 (46.53%)	13 (37.14%)	
**ECOG score**	0	87 (63.97%)	64 (63.37%)	23 (65.71%)	1.0000
	1	46 (33.82%)	34 (33.66%)	12 (34.29%)	
	2	2 (1.47%)	2 (1.98%)		
	3	1 (0.74%)	1 (0.99%)		
**Smoking status**	Former	37 (27.21%)	31 (30.69%)	6 (17.14%)	0.1206
	Never	99 (72.79%)	70 (69.31%)	29 (82.86%)	
**First-line TKI**	Afatinib	76 (55.88%)	59 (58.42%)	17 (48.57%)	0.0202
	Erlotinib	38 (27.94%)	31 (30.69%)	7 (20.00%)	
	Gefitinib	22 (16.18%)	11 (10.89%)	11 (31.43%)	
**Stage**	IIIB	1 (0.74%)	1 (0.99%)		
	IV	135 (99.26%)	100 (99.01%)	35 (100.0%)	
**Metastatic status**	More than one organ metastasized	65 (47.79%)	51 (50.50%)	14 (40.00%)	0.2841
	Only one organ metastasized	71 (52.21%)	50 (49.50%)	21 (60.00%)	
**Tissue EGFR mutation detail**	E19Del	54 (39.71%)	43 (42.57%)	11 (31.43%)	0.5903
	Ex19Del,EX20INS	1 (0.74%)	1 (0.99%)		
	Ex19Del,T790M	1 (0.74%)	1 (0.99%)		
	G719X	3 (2.21%)	3 (2.97%)		
	G719X,S768I	2 (1.47%)	2 (1.98%)		
	L858R	73 (53.68%)	50 (49.50%)	23 (65.71%)	
	L861Q	2 (1.47%)	1 (0.99%)	1 (2.86%)	
**Objective response rate**		55.15% (75/136)	60.40% (61/101)	40.00% (14/35)	
**Clinical benefit rate**		96.32% (131/136)	96.04% (97/101)	97.14% (34/35)	
**Median PFS (months)**		13.1	11.5	17.5	0.0072

Objective response rate = (CR+PR)/Total

Clinical benefit rate = (CR+PR+SD)/Total.

**Fig 2:**
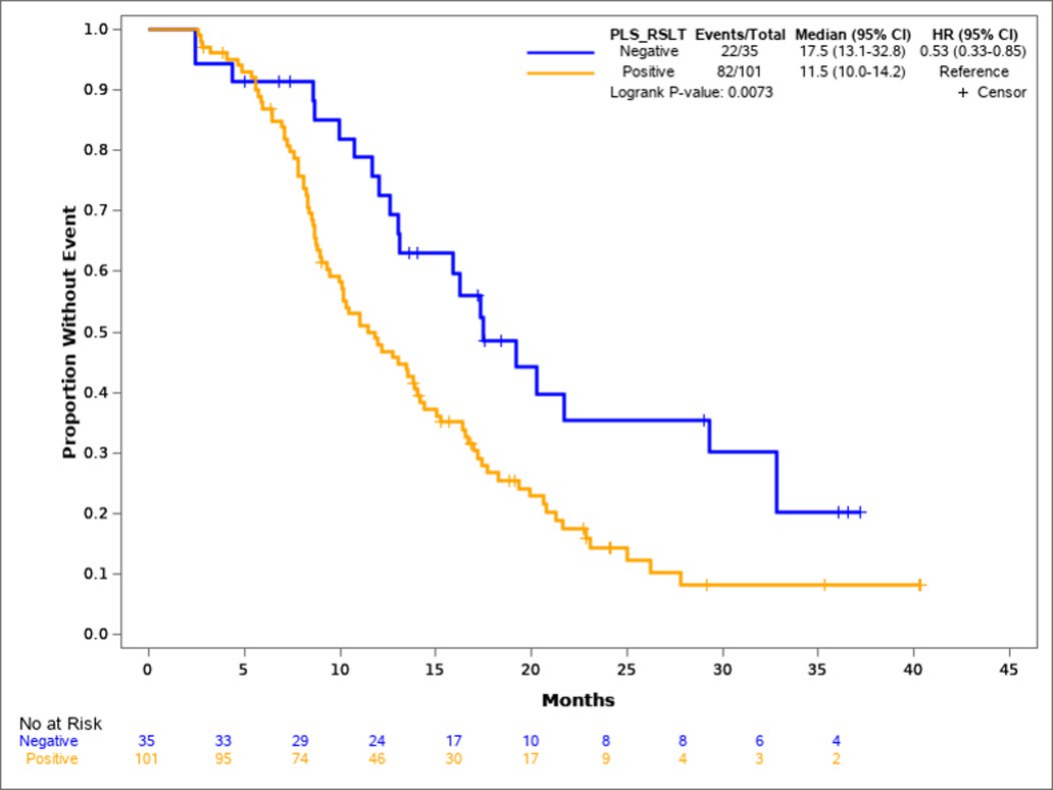
Kaplan-Meier survival analysis of plasma EGFR mutation positive and negative patients. There were 22 events in the 35 patients with negative plasma EGFR mutation, and 82 events in the 101 patients who were plasma EGFR mutation positive. The median PFS was 17.5 months and 11.5 months for the plasma EGFR mutation negative and positive population, respectively, with a HR of 0.53 and significant p value.

The median PFS of the 136 patients was 13.1 months, which is in line with what has been reported in the literature [16, 17]. It has also been reported that patients who are EGFR tissue positive/plasma negative have better survival compared with those who are both EGFR tissue and plasma positive [8, 18]. Our findings were consistent with this observation as the median PFS of patients who were EGFR tissue and plasma positive was 11.5 months compared with the 17.5 months in patients who were EGFR tissue positive/plasma negative (HR: 0.53, p = 0.0073, Fig 2).

### Comparison between tissue and plasma EGFR mutation testing result

We examined the concordance between blood and tissue samples at baseline in this Taiwanese population. There were 199 baseline paired plasma and tissue samples (of the 231 screened patients) that were tested with the cobas EGFR Test. The positive and negative percent agreement between tissue and plasma EGFR variant detection was 74.5% and 90.5%, respectively (S1 Table), which is consistent with published data [8, 18, 19]. There were more patients who were plasma-positive with multi-organ involvement as compared with plasma-negative patients (Table 1), consistent with a higher tumor burden in plasma-positive patients.

### Response predictive power of cfDNA EGFR mutations

**Fig 3:**
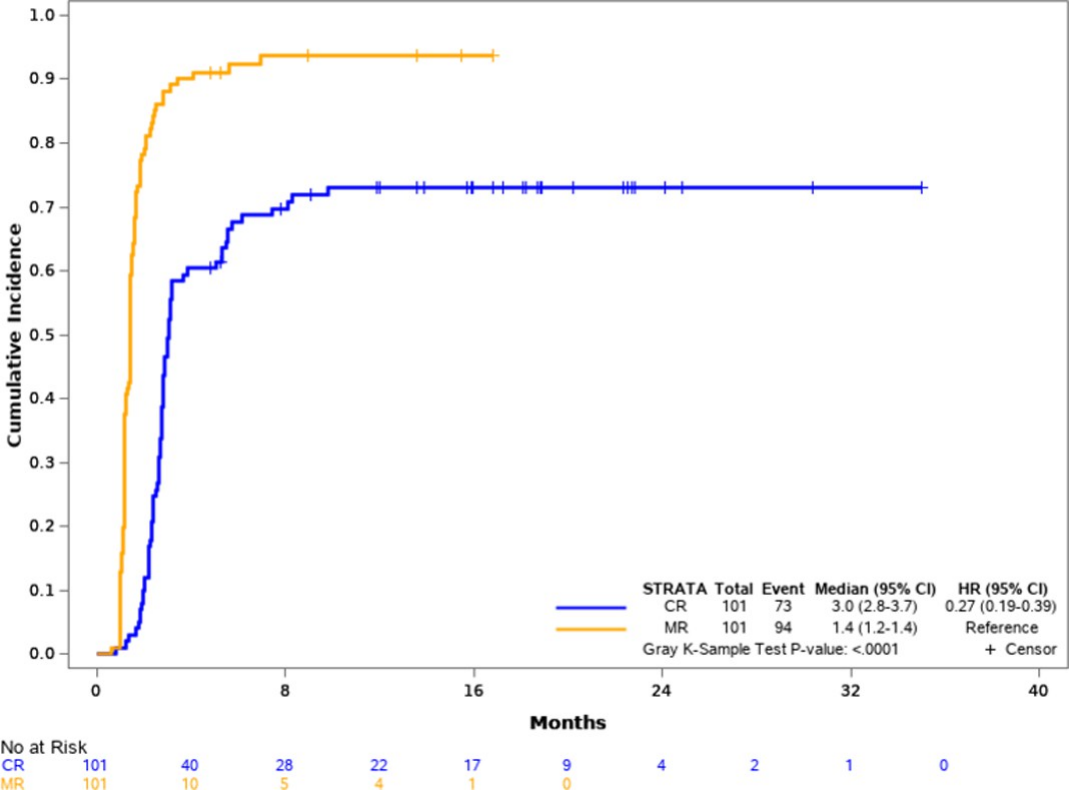
Cumulative incidence analysis of molecular and clinical response. The time to response was estimated; the median time to molecular response was 1.4 months and the median time to clinical response was 3.0 months.

A cumulative incidence approach was used to explore the relationship between clinical response (complete response and partial response based on RECIST 1.1) and molecular response, which was defined as a result of plasma EGFR mutation negative in patients who were originally plasma EGFR mutation positive; in the 101-patient population, 73 patients (72%) had a RECIST-defined clinical response and 94 patients (93%) had a molecular response. As shown in Fig 3, the median time to molecular response was 1.4 months and the median time to clinical response was 3.0 months. Molecular response was highly predictive of clinical response as 69/73 (95%) patients who had a clinical response also had a molecular response. In those patients who did not have a molecular response (n = 7), none had a clinical response.

### Changes in cfDNA EGFR mutations and their association with disease progression

**Fig 4:**
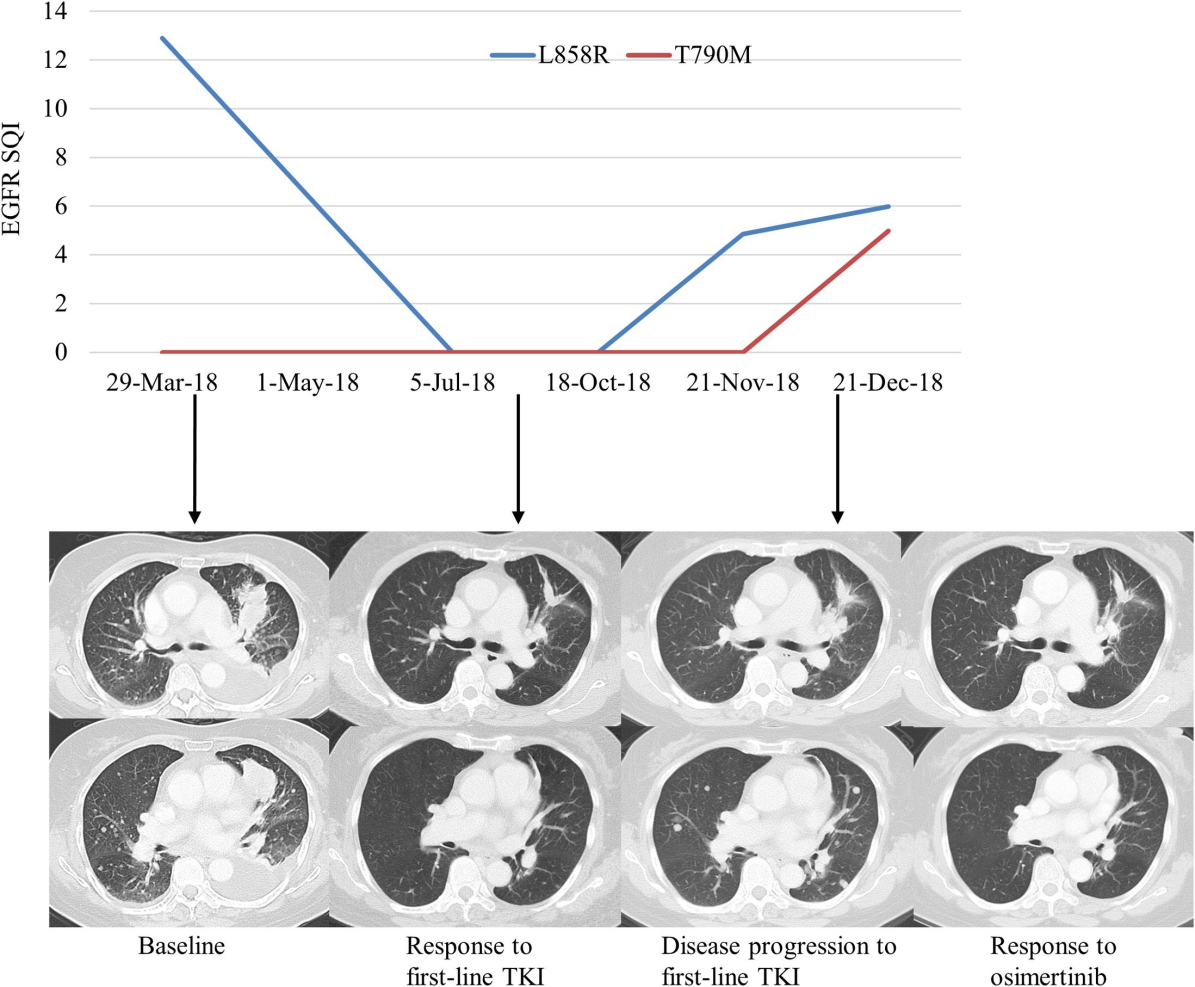
Case study of a patient with L858R and T790M mutations who progressed on first-line TKI therapy. Analysis of EGFR SQI for each mutation over time with corresponding serial chest imaging.

**Fig 5:**
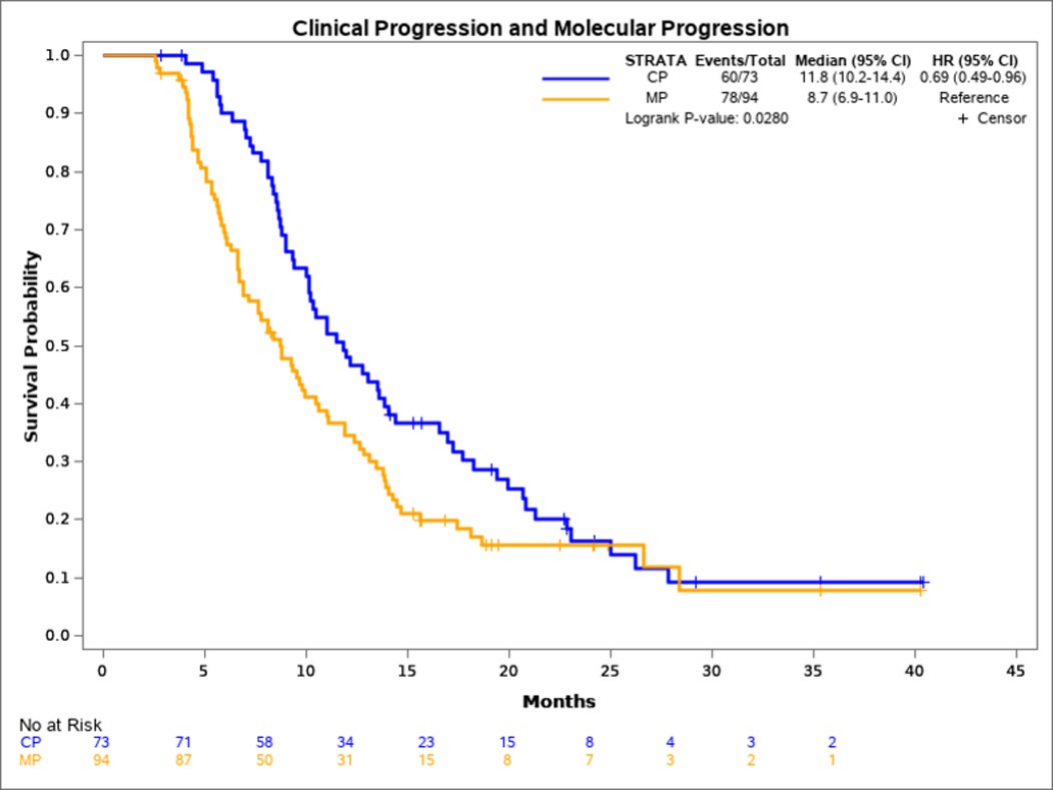
Kaplan-Meier survival analysis of patients with clinical progression versus patients with molecular progression. The median PFS of patients with molecular progression was 8.7 months vs 11.8 months for patients who clinically progressed. The HR was 0.69 with a p value of 0.0280.

To assess the potential clinical utility of monitoring EGFR mutations in plasma during first-line anti-EGFR TKI treatment, we assessed EGFR mutations in the plasma samples of 101 patients who were EGFR plasma-positive at baseline. Plasma samples were collected every 6 weeks during first-line TKI therapy until progression. At the time of this analysis, there were 58 patients who had progressed on first-line TKI therapy who had serial plasma samples available for analysis. This analysis showed the re-emergence of the original EGFR-sensitizing mutations with increasing SQI levels and/or detection of a T790M mutation. Fig 4 depicts the change in EGFR mutation load according to SQI value and serial chest imaging in a representative patient case. The differences in time to progression (molecular progression versus clinical progression) were calculated using the Kaplan-Meier method. The median time to molecular progression was 8.7 months vs 11.8 months for clinical progression (Fig 5). The median time difference between molecular progression and clinical progression was 3.1 months with a log-rank p-value of 0.028.

### Changes in cfDNA EGFR mutations and their association with survival based on clinical and molecular response

We examined the correlation between molecular progression and clinical progression for the 69 patients who had both a molecular and clinical response. There were 56 patients who clinically progressed, of whom 53 (94.6%) also had molecular progression (Table 2). The mean time difference between clinical progression and molecular progression in this subgroup of 53 patients was 3.9 months (S2 Table). Interestingly, of the three patients who clinically progressed without molecular progression (S3 Table), all progressed based on the development of new central nervous system (CNS)/brain metastases.

**Table 2:** Progression comparison for patients who were both clinical and molecular responders.

	Clinical progression		
Molecular progression	Yes	No	Total
**Yes**	53	5	58
**No**	3	8	11
**Total**	56	13	69

## Discussion

This study was a prospective, observational study at a single site in Taiwan to evaluate the feasibility of using liquid biopsies to monitor patients for treatment response and disease progression. Blood-based testing compared with tissue testing for EGFR activating mutations had a positive percent agreement of 74.5% and a negative percent agreement of 90.5%, with a 78% overall percentage agreement between matched plasma and tumor tissue samples. This is in line with other published data [8, 19, 20] and further demonstrates the utility of liquid biopsy in EGFR-positive NSCLC patient management. Although the concordance between tissue and plasma was relatively good, the specificity in our analysis was not as high as others have reported [21]. This may be due to the observational nature of this study. The study population was also biased towards EGFR mutation positive patients, with only 42 (21%) EGFR tissue negative patients included in the analysis.

The disappearance of EGFR mutations from plasma was highly correlated with clinical response. 69 of 73 patients (94.5%) who received first-line anti-EGFR treatment responded clinically and had a decrease in their plasma EGFR mutation load, which was defined as a molecular response. ctDNA has emerged as an increasingly accepted alternative approach in the area of clinical monitoring, therapy response prediction, minimum residual disease assessment, and early cancer detection [14, 22–25]. In Taiwan, the first imaging in routine clinical practice to assess response or progression on treatment is 2 to 3 months from the start of therapy. In this study, the first blood collection after treatment start was taken at about 6 weeks. Based on the high correlation between molecular response and clinical response, the analysis of plasma sample at the 6-week time point was able to provide an early indication of patient response to anti-EGFR therapy.

Likewise, the reappearance of the original EGFR-sensitizing mutation and/or the detection of a new T790M mutation was highly correlated with clinical progression. The lead time provided by detecting molecular progression could allow the physician to monitor the patient more closely and be prepared to change therapies upon clinical progression. For those patients who develop a treatment-emergent T790M mutation, changing therapy to a third-generation EGFR TKI with activity against T790M, such as osimertinib, is likely to provide further long-term control of the patient’s disease. However, this would require a randomized trial to confirm such a strategy. Of note, there were three patients who had clinical disease progression without molecular progression and all three patients progressed with new metastatic tumors in central nervous system (CNS)/brain. Not detecting molecular progression (increase of the target EGFR mutations or appearance of T790M) in these patients may be because the blood‐brain barrier prevents cfDNA from brain lesions from passing into the blood circulation. Any cfDNA found in the peripheral blood would most likely reflect ctDNA shed from the primary tumor and/or metastases at other extracranial tumor sites [26]. Therefore, in patients with brain metastases, cerebrospinal fluid ctDNA might be a better source of determining the molecular status of intracranial lesions.

Finally, as observed by other investigators, EGFR plasma positivity at baseline is a relatively poor prognostic sign, as those patients who are EGFR tissue and plasma positive have a shorter PFS compared with patients who are EGFR tissue positive/plasma negative. This correlation may be due to a higher tumor burden in those patients who are plasma positive and are likely to progress sooner.

This study has several limitations. This was a real-world study with patients treated at a single hospital in Taiwan. The patients selected for this analysis were required to have at least two follow-up plasma samples collected. This may bias the sample set with patients who had better survival and would miss patients with early disease progression. The blood collections were not concurrent with CT imaging and may bias the evaluations of time to response and progression. Brain imaging was not performed routinely in this study; some patients with CNS progression were detected during image follow-up and some based on new CNS symptoms. Other resistance mechanisms of the patients with CNS progression were not identified as biopsy of CNS lesions is not feasible, and previous research has shown that plasma-based liquid biopsy has limited performance in patients with NSCLC and isolated CNS progression [27].

## Conclusion

This study demonstrated that monitoring EGFR mutation levels or changes in blood could be a meaningful approach to predict clinical response and progression for lung EGFR mutation-positive adenocarcinoma patients treated with TKI therapy. Using liquid biopsy to confirm response or lack of response could lead to new treatment strategies to optimize the patient care continuum. It could also provide the clinician the ability to predict progression and provide lead time to strategize about further therapy. Further studies are warranted to demonstrate the potential clinical utility of serial blood EGFR testing in NSCLC management.

## Supporting information

S1 Checklist Inclusivity in global research. (DOCX)

S1 Table Agreement between plasma and tissue mutation results at baseline. (DOCX)

S2 Table Median and mean for clinical and molecular progression-free survival. (DOCX)

S3 Table Listing of the three patients who were clinically progressed but not molecularly progressed. (DOCX)
