# Supplementary material for: Efficacy of liquid biopsy for disease monitoring and early prediction of tumor progression in EGFR mutation-positive non-small cell lung cancer
Source: PLoS One. 2022 Apr 28;17(4):e0267362. doi: 10.1371/journal.pone.0267362 (PMC9049536; doi:10.1371/journal.pone.0267362)
Supplement: S1 Table — (DOCX) [file pone.0267362.s002.docx]

**S1 Table. Agreement between Plasma and Tissue Mutation Results at Baseline.**

|  | **Tumor Tissue EGFR Mutation** | | |  |
| --- | --- | --- | --- | --- |
| **Plasma EGFR mutation** | **Positive** | **Negative** | **Missing** | **Total** |
| **Positive** | 117 | 4 | 13 | 134 |
| **Negative** | 40 | 38 | 16 | 94 |
| **Missing** | 3 | - | - | 3 |
| **Total** | 160 | 42 | 29 | 231 |
| **Positive percent agreement**  **(sensitivity) (95%CI)** | 74.5% (117/157) (67.2%, 80.7%) | - | - | - |
| **Negative percent agreement**  **(specificity) (95%CI)** | 90.5% (38/42)  (77.9%, 96.2%) | - | - | - |
| **Positive predictive value**  **(95%CI)** | 96.7% (117/121)  (91.8%, 98.7%) | - | - | - |
| **Negative predictive value**  **(95%CI)** | 48.7% (38/78)  (37.9%, 59.6%) | - | - | - |
