# Supplementary material for: Efficacy of liquid biopsy for disease monitoring and early prediction of tumor progression in EGFR mutation-positive non-small cell lung cancer
Source: PLoS One. 2022 Apr 28;17(4):e0267362. doi: 10.1371/journal.pone.0267362 (PMC9049536; doi:10.1371/journal.pone.0267362)
Supplement: S2 Table — (DOCX) [file pone.0267362.s003.docx]

**S2 Table. Median and Mean for Clinical and Molecular Progression-Free Survival.**

|  | **N** | **Median (months)** | **Mean (months)** |
| --- | --- | --- | --- |
| **Clinical progression duration** | 53 | 11.0 | 12.7 |
| **Molecular progression duration** | 53 | 7.6 | 8.8 |
