# Supplementary material for: Efficacy of liquid biopsy for disease monitoring and early prediction of tumor progression in EGFR mutation-positive non-small cell lung cancer
Source: PLoS One. 2022 Apr 28;17(4):e0267362. doi: 10.1371/journal.pone.0267362 (PMC9049536; doi:10.1371/journal.pone.0267362)
Supplement: S3 Table — (DOCX) [file pone.0267362.s004.docx]

**S3 Table. Listing of the Three Patients who were Clinically Progressed but not Molecularly Progressed.**

| **Patient ID** | **Enroll ID** | **CT Scan Visit Date** | **CT Scan Visit Description** | **Lesion Description** | **Lesion Diameter** | **First Clinical Progression Date** |
| --- | --- | --- | --- | --- | --- | --- |
| **S080** | E065 | 04FEB2017 | Baseline | lung tumor | 31 |  |
|  | E065 | 18MAY2017 | Visit 1 | lung tumor | 17 |  |
|  | E065 | 16AUG2017 | Visit 2 | lung tumor | 17 |  |
|  | E065 | 24SEP2017 | Visit 3 | brain mets | N.A | 24SEP2017 |
| **S119** | E082 | 05JUL2017 | Baseline | lung tumor | 43 |  |
|  | E082 | 11OCT2017 | Visit 1 | lung tumor | 30 |  |
|  | E082 | 16JAN2018 | Visit 2 | lung tumor | 30 |  |
|  | E082 | 12APR2018 | Visit 3 | CSF tumor seedings | N.A | 12APR2018 |
| **S144** | E098 | 17OCT2017 | Baseline | lung tumor | 32 |  |
|  | E098 | 05FEB2018 | Visit 1 | lung tumor | 18 |  |
|  | E098 | 11MAY2018 | Visit 2 | lung tumor | 18 |  |
|  | E098 | 10AUG2018 | Visit 3 | lung tumor | 20 |  |
|  | E098 | 05NOV2018 | Visit 4 | lung tumor | 20 |  |
|  | E098 | 25DEC2018 | Visit 5 | brain mets | 15 | 25DEC2018 |
